# The International Trauma Questionnaire Child and Adolescent Version (ITQ-CA) in Portuguese: validation for children at risk

**DOI:** 10.1080/20008066.2026.2638114

**Published:** 2026-03-17

**Authors:** André Moreira, Flávia Osório, José Pacheco, Ana Moura, Margarida Rangel Henriques, José Carlos Rocha

**Affiliations:** a Universidade do Porto, Porto, Portugal; b Universidade de São Paulo, Ribeir˜o Preto, Brazil; c Rede Unidas, Comunidade Intermunicipal do Tâmega e Sousa, Penafiel, Portugal; d Associate Laboratory i4HB - Institute for Health and Bioeconomy, University Institute of Health Sciences - CESPU, Gandra, Portugal

**Keywords:** ITQ-CA, complex PTSD, PTSD, residential care, childhood trauma, ITQ-CA, trastorno por estrés postraumático complejo, trastorno por estrés postraumático, acogimiento residencial, trauma infantil

## Abstract

**Background:** Children and adolescents exposed to adversities, such as those in residential care or those affected by domestic violence (DV) but not in care, experience elevated rates of trauma, placing them at risk for both posttraumatic stress disorder (PTSD) and complex PTSD (CPTSD).

**Objective:** Our objective is to validate the Portuguese adaptation of the International Trauma Questionnaire – Child and Adolescent Version (ITQ-CA) to assess for ICD-11 PTSD and CPTSD in at-risk Portuguese-speaking youths.

**Methods:** The ITQ-CA was translated and culturally adapted following established guidelines. This study included 160 participants aged 7–17 years, comprising children in residential care and those exposed to DV. Confirmatory factor analyses (CFA) were conducted to test competing ICD-11 consistent latent structural models of PTSD and disturbances in self-organisation (DSO). Convergent validity was examined using the CRIES-13 and ITEM-CA, and divergent validity was assessed using the WHO-5. Exploratory factor analysis and network analysis were conducted as supplementary analyses and are reported in the Supplementary Materials.

**Results**: CFA supported the two higher order correlated factors model with two-factor higher-order (PTSD and DSO), yielding adequate fit indices, *χ*^2^(52) = 104.189, RMSEA = 0.079, CFI = 0.923, TLI = 0.903. Internal consistency is strong (ω = 0.872; α = 0.871). The ITQ-CA demonstrated significant correlations with the CRIES-13 and ITEM-CA, supporting convergent validity, while negative correlations with the WHO-5 confirmed divergent validity.

**Conclusion:** The Portuguese version of the ITQ-CA demonstrated strong psychometric properties, supporting its use as a valid and reliable tool for identifying PTSD and CPTSD symptoms in children and adolescents. Its integration into practice could support targeted trauma-informed interventions.

## HIGHLIGHTS

- The ITQ-CA was successfully adapted and validated for use with Portuguese-speaking children and adolescents.
- Results confirmed strong psychometric properties, supporting its reliability and validity in identifying PTSD and complex PTSD symptoms.
- The ITQ-CA offers clinicians a culturally adapted, evidence-based tool to guide trauma-informed care for Portuguese-speaking youth.

## Abstract (es)

**Antecedentes:** Los niños y adolescentes expuestos a adversidades, como aquellos en acogimiento residencial o afectados por violencia doméstica (VD) sin estar institucionalizados, presentan tasas elevadas de trauma, lo que los sitúa en riesgo tanto de trastorno por estrés postraumático (TEPT) como de trastorno por estrés postraumático complejo (TEPT-C).

**Objetivo:** Nuestro objetivo es validar la adaptación portuguesa del Cuestionario Internacional de Trauma – Versión para Niños y Adolescentes (ITQ-CA, por sus siglas en inglés) para evaluar TEPT y TEPTC según la CIE-11 en jóvenes lusohablantes (*) en situación de riesgo.

**Métodos:** El ITQ-CA fue traducido y adaptado culturalmente siguiendo directrices establecidas. Este estudio incluyó 160 participantes de entre 7 y 17 años, compuestos por niños en acogimiento residencial y aquellos expuestos a VD. Se realizaron análisis factoriales confirmatorios (AFC) para contrastar modelos estructurales latentes alternativos consistentes con la CIE-11 de TEPT y alteraciones en la autoorganización (DSO, por sus siglas en inglés). La validez convergente se examinó mediante el CRIES-13 y el ITEM-CA, y la validez divergente se evaluó utilizando el WHO-5. Como análisis complementarios se llevaron a cabo análisis factorial exploratorio y análisis de redes, cuyos resultados se presentan en los Materiales Suplementarios.

**Resultados:** El AFC respaldó el modelo de dos factores correlacionados de orden superior, con dos factores de orden superior (TEPT y DSO), mostrando índices de ajuste adecuados, χ² (52) = 104.189, RMSEA = 0.079, CFI = 0.923, TLI = 0.903. La consistencia interna es alta (ω = 0.872; α = 0.871). El ITQ-CA mostró correlaciones significativas con el CRIES-13 y el ITEM-CA, apoyando la validez convergente, mientras que las correlaciones negativas con el WHO-5 confirmaron la validez divergente.

**Conclusión:** La versión portuguesa del ITQ-CA demostró sólidas propiedades psicométricas, lo que respalda su uso como una herramienta válida y fiable para identificar síntomas de TEPT y TEPTC en niños y adolescentes. Su integración en la práctica podría favorecer intervenciones orientadas a trauma y dirigidas específicamente a esta población.

(*) Nota del traductor: forma equivalente a hispanohablante, para referirse a las personas que hablan portugués nativamente.

## 1. Background

Trauma exposure in children and adolescents is a critical global public health issue, particularly among vulnerable populations such as those in foster or residential care or those exposed to domestic violence (DV). These groups frequently experience multiple types of trauma, including neglect, physical abuse, emotional abuse, and exposure to caregiver substance abuse, both before and during placement, with children in foster care exposed to an average of 4.7 traumatic events (Beyerlein et al., 2019; Forkey et al., 2016; Greeson et al., 2011; Lehmann et al., 2020; Reddy et al., 2013). Children in residential care face even higher exposure, averaging 5.8 events (Briggs et al., 2012; Collin-Vézina et al., 2011). These adversities result in significant trauma-related symptoms and impaired social and educational functioning. Prevalence estimates of trauma symptoms among children in care vary widely, from 6.6% to 70%, reflecting differences in definitions, populations, and assessment methods. Exposure to DV is also strongly correlated with PTSD, with trauma symptoms often emerging within weeks of exposure (Bogat et al., 2006; Levendosky et al., 2002; Wherry et al., 2015).

Trauma exposure and PTSD are linked to adverse outcomes such as poorer physical and behavioural health, increased aggression, internalising symptoms, and substance use (Beal et al., 2019; Farley et al., 2022; Haselgruber et al., 2020; Villodas et al., 2016). Adverse childhood experiences (ACEs) are traumatic events occurring before age 18, including abuse, neglect, and family dysfunction. Exposure to multiple ACEs predicts adverse physical and mental health outcomes across the lifespan, such as obesity, depression, and suicidality (Asmussen et al., 2020; Felitti et al., 1998). Children in foster care are disproportionately affected by ACEs compared to general population peers (Turney & Wildeman, 2017). Higher ACE exposure increases the likelihood of homelessness, depressive symptoms, and engagement in problem behaviours in adulthood (Rebbe et al., 2017). These findings underscore the cumulative impact of early adversity and the importance of accurate assessment in at-risk youth.

CPTSD, recently introduced in the ICD-11 (World Health Organization [WHO], 2018), extends the PTSD framework by including Disturbances in Self-Organisation (DSO) – affective dysregulation, negative self-concept, and relational disturbances. CPTSD often arises from prolonged or repeated trauma and is associated with more severe impairment (Cloitre et al., 2019). Youths with CPTSD frequently display emotional and cognitive difficulties, dissociation, avoidance, and problems maintaining relationships (WHO, 2018). Assessment in this population is complex due to symptom overlap with disorders such as depression, ADHD, and autism, often leading to misdiagnosis (Boodoo et al., 2022; Carpita et al., 2024; Ford & Courtois, 2021; Gilbar, 2020). Many available instruments lack specificity, requiring multiple tools to capture full symptom profiles (Hébert & Amédée, 2020; Kozlowska, 2020).

Accurate, culturally sensitive tools are essential for reliable trauma assessment. Instruments must capture both PTSD and CPTSD dimensions, align with ICD-11 diagnostic models, and ensure linguistic and cultural relevance. The International Trauma Questionnaire (ITQ) developed by Cloitre et al. (2018) and validated in Portuguese by Rocha et al. (2020) assesses PTSD and CPTSD in adults. Its adaptation for younger populations, the International Trauma Questionnaire – Child and Adolescent Version (ITQ-CA), was validated in German by Haselgruber et al. (2020) and demonstrated strong factorial and construct validity. However, a validated Portuguese version for children and adolescents has been lacking.

Recent studies have confirmed the psychometric robustness of the ITQ-CA across several linguistic and cultural contexts. The original German validation demonstrated a two-factor higher-order structure distinguishing PTSD and DSO in foster children (Haselgruber et al., 2020). A Chinese validation in a clinical adolescent sample replicated this model and showed strong reliability and good fit indices (Ho et al., 2022). In a Danish sample of abuse-exposed youths, Løkkegaard et al. (2023) found the same two-factor solution and supported measurement invariance across gender. Similarly, the Farsi version demonstrated excellent internal consistency and factorial validity in Iranian children and adolescents exposed to trauma (Parhoon et al., 2024). More recently, the Greenlandic adaptation confirmed the cross-cultural stability of the ITQ-CA in an Indigenous adolescent population (Banzon et al., 2024). Collectively, these validations support the ITQ-CA as a culturally adaptable and psychometrically sound instrument, underscoring the importance of validating a Portuguese version to extend its applicability to Lusophone contexts.

We aim to validate the Portuguese version of the ITQ-CA in a sample of at-risk youths in residential care or exposed to DV. We expect a two-factor structure consistent with PTSD and DSO, strong internal consistency, and evidence of convergent, and divergent validity.

## 2. Methods

### 2.1. Translation and adaptation

Translation and adaptation followed internationally recognised guidelines (International Test Commission, 2018; Sousa & Rojjanasrirat, 2011). Two translations were produced independently: one by the first author and one by a trauma specialist fluent in English and Portuguese. Translations were reconciled, back-translated, and finalised in consultation with the original ITQ-CA authors.

### 2.2. Participants and procedures

Children from residential care institutions and children exposed to DV were invited to participate. A total of 160 participants with a mean age of 12.16 years (*SD* = 2.90), comprised of 39.5% boys and 60.5% girls, accepted. Of the total, 56 participants were recruited from residential care facilities and 104 were referred to the Portuguese child protection services ‘*Comissão de Proteção de Crianças e Jovens*’ (CPCJ) in the region of the Intermunicipal Community of Tâmega e Sousa, after being victims of DV or being exposed to DV. To be included, the children/youths had to have been in residential care or have been exposed to DV and be 7–17 years old. Cases identified with cognitive deficit were not included based on institutional information (formal neurodevelopmental/psychological assessment or clinical diagnosis recorded by a psychologist or child psychiatrist).

Ethical approval was obtained from the Faculty of Psychology and Education Sciences of the University of Porto Ethics Board (process no. 2022/07-01). Written informed consent from legal guardians/tutors (e.g. parents, appointed legal guardians, or the responsible child-protection authority) and written assent from children/adolescents were collected prior to participation. Consent was obtained by trained study personnel (licensed psychologists or designated social workers employed by the residential care institution or child protection service) who explained study aims, confidentiality, and the voluntary nature of participation. For participants recruited via child protection services (CPCJ), consent was provided by the child’s legal tutor or by CPCJ when legal guardianship was held by the service. Data was collected by trained psychologists in a private setting within the residential facility or at the referring service; staff members were proximally available but did not sit with the child during completion to preserve confidentiality.

### 2.3. Measures

Assessment instruments are presented in order of application.

The *International Traumatic Exposure Measure – Child and Adolescent Version* (ITEM-CA) is a self-report instrument designed to assess the level of children and adolescents between 7- and 18-years old exposure to adverse and traumatic events (α = .88), developed by Rocha et al. (2023). This instrument has 38 items, of which two are not specific regarding traumatic exposure but question the potential need for professional help to overcome difficulties and include an ‘exit item’ about happy moments in the child/adolescent’s life. It also includes four complementary items to assess which of the traumatic events was the worst (item 1), how many times it happened (item 2), how long ago (item 3), and the emotions associated with the traumatic event (item 4). In our sample, this instrument had an internal consistency of α = .90.

The ITQ-CA (Haselgruber et al., 2020) was designed to assess PTSD and CPTSD in children between 7 and 17 years old, which this study focused on validating. It is a self-report measure with 22 items. Prior validations have reported acceptable to strong internal consistency for the ITQ-CA (e.g. Haselgruber et al., 2020; Ho et al., 2022; Parhoon et al., 2024), with alpha coefficients ≥.83. The participant chooses the most impactful event, based on the questionnaire used to assess traumatic exposure (ITEM-CA), and answers the questions of the ITQ-CA with this event in focus. Six items are used to assess PTSD, with two items assessing *re-experiencing* (Re1 and Re2), two items assessing *avoidance* (Av1 and Av2), and two items assessing *threat perceptions* (Th1 and Th2). Six other items are used to assess DSO symptoms: *affective dysregulation* (AD1 and AD2), *negative self-concept* (NSC1 and NSC2), and *disturbances in relationships* (DR1 and DR2). The ITQ-CA includes a 5-point frequency Likert scale from 0 ‘never’ to 4 ‘almost always’, assessing how much the participants have been bothered by the symptoms over the last month. A score ≥ 2 indicates the presence of symptoms. Besides these 12 items, 10 extra items are used to assess functional impairment related to both PTSD and DSO symptoms in occupational, educational, social, familial, and other vital areas of functioning as well as general happiness. Impairment items are answered on a binary scale (yes or no). Symptom items on the ITQ-CA comprise 12 items rated 0 (‘never’) to 4 (‘almost always’), yielding a symptom score range of 0-48; higher scores indicate greater PTSD/CPTSD symptom severity. The possible score range for each subscale is 0–24. Functional impairment items are binary and reported separately. This instrument is available at: https://www.traumameasuresglobal.com/_files/ugd/be25b4_4a05f7b652a04a998af578c06e3e90d5.pdf.

The *Children Revised Impact of Events Scale* (CRIES-13) (Children and War Foundation, 2005) is a self-report measure with 13 items that assesses PTSD in children and adolescents between 7 and 17 years old. Of these, four items measure *intrusion*, four measure *avoidance*, and five measure *arousal*. This scale was translated into Portuguese by Rocha et al. (2012), with an α = .83, and an internal consistency of α = .82 was obtained in our sample. This questionnaire was then used to assess convergent validity.

The *World Health Organisation Five Well-Being Index* (WHO-5) is a self-report measure of mental well-being that was used to assess divergent validity. It consists of five statements relating to the past 2 weeks, with an average of α = .84 across different studies and populations. Statements are rated on a 6-point scale, with higher scores indicating better well-being (Topp et al., 2015; WHO, 1998). In our sample, the WHO-5 showed an internal consistency of α = .78.

### 2.4. Data analysis

Descriptive statistics (means, standard deviations, and frequencies) are computed for demographic variables and questionnaire scores. Internal consistency for the ITQ-CA total and subscales is evaluated with Cronbach’s α and McDonald’s ω to ensure a robust assessment of reliability.

An exploratory factor analysis with principal axis factoring and varimax rotation, was performed to screen for sample-specific anomalies or extreme cross-loadings, and determination of the number of factors through parallel analysis; detailed results are reported in the Supplementary Materials and are not used as evidence for model selection. Next, confirmatory factor analyses (CFA) are performed to test four theoretically grounded models based on previous ITQ-CA studies: (1) a single-factor CPTSD model, (2) six correlated first-order factors, (3) a single higher-order CPTSD factor, and (4) two correlated higher-order factors (PTSD and DSO). Models are estimated using robust maximum likelihood (MLR) with full information maximum likelihood for missing data. Model fit is evaluated with χ², RMSEA (≤.08 acceptable, ≤.05 good), CFI and TLI (≥.90 acceptable, ≥.95 excellent), and BIC for model comparison (Hu & Bentler, 1999; Kline, 2016).

In the present study, confirmatory factor analysis was used to test whether the Portuguese ITQ-CA conforms to the ICD-11 latent structure of PTSD and DSO. Network analysis using a Gaussian Graphical Model with regularisation (EBICglasso) is also included in Supplementary Materials as an exploratory, descriptive procedure to visualise patterns of symptom co-occurrence at the item level in this high-risk sample (Borsboom et al., 2021; Epskamp et al., 2018).

Finally, Pearson correlations examined associations between ITQ-CA PTSD, DSO, and total scores and external measures (CRIES-13, ITEM-CA, WHO-5) to evaluate convergent and divergent validity. Effect sizes followed Cohen’s (1988) benchmarks (*r* ≈.10 small, *r* ≈.30 medium, *r* ≥ .50 large).

## 3. Results

### 3.1. Descriptive statistics

Table 1 summarises the prevalence of reported adverse and traumatic events exposure. Emotional violence within the family (78.1%), separation from parents (66.9%), and exposure to violence (49.4%) are among the most frequently reported experiences. Participants reported an average of 9.03 (*SD* = 5.46) traumatic events, with 30% of participants reporting exposure to adverse and traumatic events both during childhood and adolescence. These findings reflect the cumulative and multi-type nature of adverse and traumatic events among the sampled population.The mean total ITQ-CA score is *M* = 17.34 (*SD* = 10.23). The PTSD subscale showed a mean of *M* = 9.71 (*SD* = 5.38), while the DSO subscale had a mean of *M* = 7.62 (*SD* = 5.89).

**Table 1.** Frequency of type of adverse and traumatic events experienced as assessed with the ITEM-CA (%).

Trauma exposure type	% reporting
Emotional violence within the family	78.1%
Separation from parents	66.9%
Trauma related to health (illness, exams)	65.0%
Trauma related to death of loved ones	51.2%
Exposure to violence	49.4%
Accidents (involvement or exposure)	49.0%
Severe mental illness in the family	46.9%
Emotional abuse within the family	43.2%
Parental legal involvement or incarceration	33.8%
Violence within the family	32.5%
Trauma during both childhood and adolescence	30.0%
Sexual abuse	23.1%
Exposure to or involvement in disasters	18.8%
Stalking	10.0%
Neglect (basic needs unmet)	8.8%
Other (e.g. moving, peer issues, injuries)	8.1%

### 3.2. ITQ-CA internal validity

#### 3.2.1. Confirmatory factor analysis

**Figure 1.**
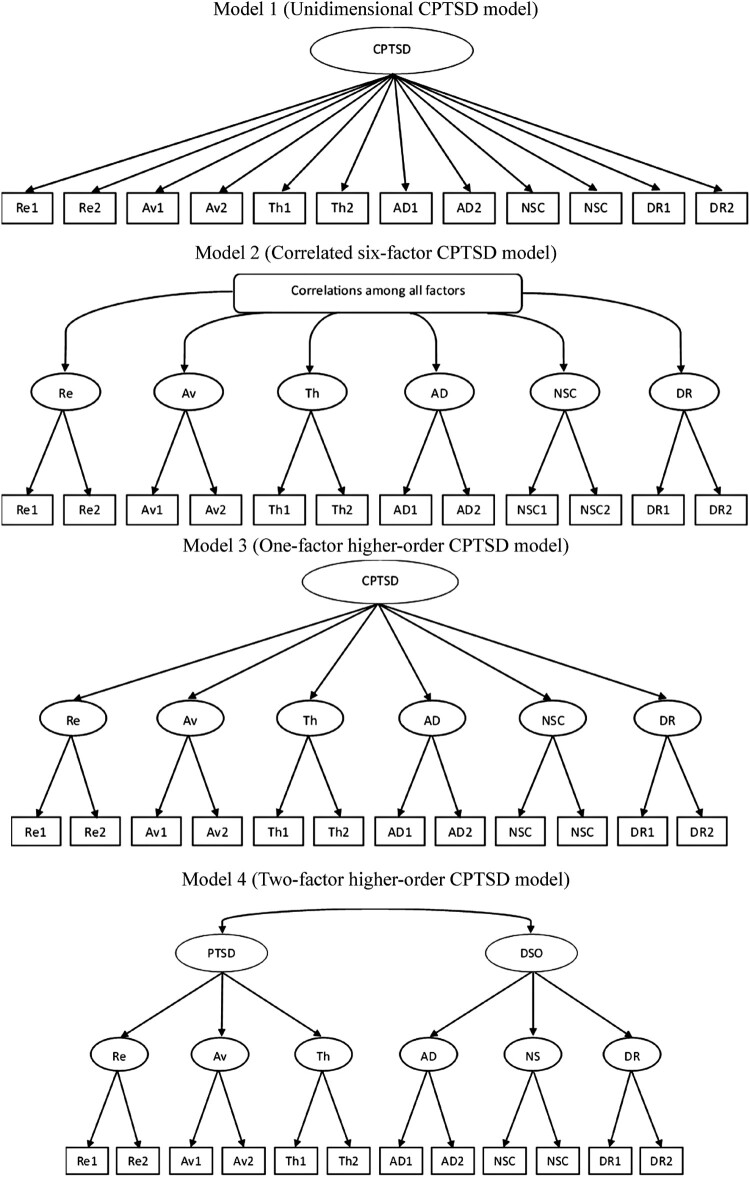
ICD-11 CPTSD tested models involving Re: re-experiencing; Av: avoidance; Th: sense of current threat; AD: affective dysregulation; NSC: negative self-concept; and DR: disturbances in relationships.

Based on previous research on the ITQ and ITQ-CA, we specified four models of ICD-11 CPTSD symptom structure in children through CFA. Model 1 represents a single-factor model with all items loading on a single latent factor (CPTSD). Model 2 is comprised of six correlated first-order factors (re-experiencing, avoidance, threat, affective dysregulation, negative self-concept, disturbances in relationships). Model 3 includes six first-order factors and one second-order factor (CPTSD). Model 4 is comprised of six first-order factors and two correlated second-order factors (PTSD, DSO), with first-order factors re-experiencing, avoidance, and threat loading onto PTSD, and first-order factors affective dysregulation, negative self-concept, and disturbances in relationships loading onto DSO (Figure 1).

Model-fit evaluation followed conventional guidelines (Hu & Bentler, 1999; Kline, 2016): RMSEA ≤.08 indicates acceptable fit, ≤.05 good fit; CFI and TLI ≥.90 acceptable, ≥.95 excellent, and Bayesian Information Criterion (BIC) differences > 10 indicate substantive model-fit improvement (Burnham & Anderson, 2004). Model 2 shows best fit values; however, Model 4 results aligned with the theoretical framework of PTSD and DSO, demonstrating adequate model fit (Table 2). Model 4 demonstrated a substantive improvement in model parsimony, with a BIC reduction exceeding 10 points relative to all alternative models, a threshold commonly interpreted as strong evidence favouring the more parsimonious model.

**Table 2.** Confirmatory factor analysis (CFA) model fit indices.

Model	*χ*^2^ (df)	RMSEA	CFI	TLI	BIC
1	144.296 (54)	0.103	0.867	0.838	266.101
2	61.108 (39)	0.06	0.968	0.945	258.949
3	119.935 (53)	0.089	0.902	0.878	246.815
4	104.189 (52)	0.079	0.923	0.903	236.143

Note: CFI and TLI ≥ 0.90 and RMSEA ≤ 0.08 indicate acceptable model fit. BIC is used for model comparison; lower values denote better fit.

### 3.3. Convergent, and divergent validity of the ITQ-CA

When assessing convergent validity, there are significant moderate correlations between ITQ-CA scales and PTSD symptom dimensions assessed with the CRIES-13, considering each dimension of both instruments. There are also significant moderate bivariate correlations between ITQ-CA and ITEM-CA scores (Table 3).

**Table 3.** Pearson correlations between the ITQ-CA subscale and CRIES-13 subscale (intrusion, avoidance, arousal, and traumatic exposure) scores, ITEM-CA dimensions, and WHO-5.

	CRIES-13				ITEM-CA	WHO-5
ITQ-CA	Intrusion	Avoidance	Arousal	Total	Traumatic exposure	Well-being
Re-experiencing	.531***	.350**	.431***	.542***	.341***	−.363**
Avoidance	.437***	.562***	.435***	.590***	.194*	−.243
Sense of current threat	.424**	.212	.594***	.515***	.442***	−.395**
Affective dysregulation	.400**	.316*	.381**	.453***	.357***	−.371**
Negative self-concept	.473***	.304*	.451***	.509***	.331***	−.581***
Disturbed relationships	.436***	.204	.368**	.418**	.304***	−.430**
PTSD	.601***	.491***	.641***	.718**	.411***	−.437**
DSO	.532***	.332*	.487***	.560***	.391***	−.564***
CPTSD	.626***	.452***	.621***	.703**	.441***	−.561**
PTSD Impact	.413**	.156	.273*	.348*	.167*	−.274**
DSO Impact	.328*	.085	.201	254	.117	−.155
CPTSD Impact	.390**	.127	.249	.317*	.150	−.226

Note: The table highlights the relationships between constructs to assess convergent and divergent validity of the ITQ-CA. Correlations indicate the degree of association between related constructs, with higher values reflecting stronger evidence of convergent and divergent validity. *p < .05, **p < .01, ***p < .00.

Regarding divergent validity, we find significant negative correlations between the ITQ-CA and WHO-5 (−0.561), showing reductions in well-being as ITQ-CA scores increased.

### 3.4. ITQ-CA internal consistency

Internal consistency is evaluated with both Cronbach’s α and McDonald’s ω, ensuring a robust evaluation of internal consistency and providing complementary insights into the reliability of the scale (Hayes & Coutts, 2020). McDonald’s ω and Cronbach’s α are .87 and .87 for the total scale, .75 and .75 for the PTSD subscale, and .85 and .85 for the DSO subscale, respectively, indicating strong reliability (Nunnally & Bernstein, 1994) consistent with validations from other languages (Haselgruber et al., 2020; Ho et al., 2022; Parhoon et al., 2024).

### 3.5. Symptom prevalence in the sample

Participants are classified as having likely PTSD or CPTSD based on meeting ICD-11 diagnostic thresholds, which include a symptom score of ≥ 2 in each required domain and reported functional impairment. Cut-offs were derived based on guidelines by Cloitre et al. (2018) and Haselgruber et al. (2020). CPTSD includes PTSD symptom clusters (re-experiencing, avoidance, threat) plus disturbances in self-organisation (DSO: affective dysregulation, negative self-concept, and interpersonal disturbance). In this sample, 40% meet criteria for likely PTSD (*M* = 9.7, *SD* = 5.37); 7.5% exhibit subthreshold DSO symptoms without meeting full PTSD criteria (*M* = 7.6, *SD* = 5.8); and 18.1% for likely CPTSD (*M* = 17.3, *SD* = 10.2). Functional impairment is reported by 40% in the social dimension, 37.5% in the occupational dimension, and 61% in the other-important-areas-of-functioning dimension.

## 4. Discussion

This study validated the Portuguese version of the International Trauma Questionnaire – Child and Adolescent Version (ITQ-CA) in a sample of children and adolescents exposed to domestic violence or living in residential care. Findings provide initial evidence that the Portuguese ITQ-CA is a reliable and valid tool for assessing ICD-11 posttraumatic stress disorder (PTSD) and complex PTSD (CPTSD) in Portuguese-speaking youth.

### 4.1. Factor structure

The confirmatory factor analyses supported a two-factor organisation broadly consistent with the ICD-11 distinction between PTSD and Disturbances in Self-Organisation (DSO). The CFA tested four competing models. Model 2, a six correlated first-order-factor structure, showed the best empirical fit. However, Model 4, comprising two correlated higher-order factors (PTSD and DSO), best represented the ICD-11 conceptual framework. Thus, while Model 4 did not produce the lowest fit indices, its theoretical coherence justified its interpretive preference, which was further supported by the substantial BIC reduction observed for Model 4, indicating superior balance between model fit and complexity. This pattern parallels findings from German, Chinese, Danish, Farsi, and Greenlandic ITQ-CA validations, which consistently identified strong correlations between PTSD and DSO but maintained their conceptual distinction (Banzon et al., 2024; Haselgruber et al., 2020; Ho et al., 2022; Løkkegaard et al., 2023; Parhoon et al., 2024).

### 4.2. Reliability, convergent, and divergent validity

Internal consistency coefficients (ω = .87; α = .87) indicated strong reliability across total, PTSD, and DSO subscales, consistent with prior ITQ-CA studies (Haselgruber et al., 2020; Ho et al., 2022; Parhoon et al., 2024). Convergent validity is demonstrated through moderate to strong positive correlations between ITQ-CA scores and trauma exposure and PTSD symptom measures (ITEM-CA, CRIES-13). Divergent validity is confirmed via negative correlations with psychological well-being (WHO-5), consistent with theoretical expectations. However, because no external DSO-specific measure is included, the current study could not directly assess convergent validity for the DSO construct. Future research should incorporate instruments that capture affect regulation, self-concept, and interpersonal functioning to more comprehensively examine the DSO domain.

### 4.3. Clinical and developmental interpretation

Approximately 40% of participants met criteria for probable PTSD and 18% for probable CPTSD, findings comparable to prior work with high-risk youth (Haselgruber et al., 2020; Hiller et al., 2021; Løkkegaard et al., 2023). The high rates of social and functional impairment highlight the clinical burden of trauma in these groups. The observed overlap between PTSD and DSO symptoms suggests that younger populations may display less differentiation between threat-related and self-organisational symptoms than adults, possibly due to ongoing emotional and cognitive development (Cloitre et al., 2018). Clinically, the ITQ-CA can support early identification of these mixed symptom presentations and guide individualised interventions that address both fear-based and self-concept disturbances.

### 4.4. Limitations

Some limitations must be acknowledged. The absence of an external measure of disturbances in self-organisation (DSO) restricted the examination of convergent validity for this construct. Finally, the cross-sectional design did not allow assessment of test – retest reliability or sensitivity to change, and longitudinal data is needed to evaluate the temporal stability of the Portuguese ITQ-CA.

### 4.5. Implications and future directions

The Portuguese ITQ-CA fills a significant gap by providing a culturally adapted and psychometrically supported instrument for assessing ICD-11 PTSD and CPTSD in children and adolescents. It can assist clinicians and researchers in differentiating PTSD and DSO symptom patterns, guiding treatment decisions such as prioritising affect-regulation and interpersonal-functioning interventions. Future studies should use larger, more diverse samples such as non-clinical samples also to evaluate measurement invariance across gender, age, and trauma type, and employ longitudinal designs to establish test – retest reliability and sensitivity to therapeutic change.

## 5. Conclusion

Our sample is the first evidence of the validity and reliability of the Portuguese ITQ-CA. Although the six correlated first-order model yielded the best empirical fit, the two higher-order correlated-factor model provides the most conceptually faithful representation of ICD-11 PTSD and DSO constructs. Overall, the Portuguese ITQ-CA demonstrates sound psychometric performance and extends the reach of trauma assessment tools for Portuguese-speaking children and adolescents.

## Supplementary Material

Supplementary Material.docx

## Data Availability

The data that support the findings of this study are available from the corresponding author upon reasonable request.
