# Supplementary material for: The International Trauma Questionnaire Child and Adolescent Version (ITQ-CA) in Portuguese: validation for children at risk
Source: Eur J Psychotraumatol. 2026 Mar 17;17(1):2638114. doi: 10.1080/20008066.2026.2638114 (PMC12997481; doi:10.1080/20008066.2026.2638114)
Supplement: Supplementary Material.docx [file ZEPT_A_2638114_SM4236.docx]

***Exploratory Factor Analysis***

To validate the ITQ-CA, we conducted an exploratory factor analysis (EFA) (Table), revealing a two-factor aligning with PTSD and DSO constructs. It is possible to observe double-saturation of both item 5 (Th1) and item 7 (AD1). These items are related to hypervigilance and affective dysregulation, concepts directly related, which might explain the overlap of saturation. To confirm these results and to assess the adequacy to theoretical models, we conducted a confirmatory factor analysis.

**Table.**

*Exploratory factor loadings (varimax rotation)*

|  | | | | | | | |
| --- | --- | --- | --- | --- | --- | --- | --- |
|  | | Factor 1 | | Factor 2 | | Uniqueness | |
| Re1 |  | 0.410 |  |  |  | 0.701 |  |
| Re2 |  | 0.452 |  |  |  | 0.621 |  |
| Av1 |  | 0.540 |  |  |  | 0.707 |  |
| Av2 |  | 0.628 |  |  |  | 0.557 |  |
| Th1 |  | 0.354 |  | 0.303 |  | 0.783 |  |
| Th2 |  | 0.600 |  |  |  | 0.452 |  |
| AD1 |  | 0.429 |  | 0.417 |  | 0.643 |  |
| AD2 |  |  |  | 0.626 |  | 0.424 |  |
| NSC1 |  |  |  | 0.757 |  | 0.341 |  |
| NSC2 |  |  |  | 0.733 |  | 0.414 |  |
| DR1 |  |  |  | 0.644 |  | 0.567 |  |
| DR2 |  |  |  | 0.632 |  | 0.567 |  |
| *Note.* Varimax rotation applied. Factor 1 reflects PTSD-related symptoms; Factor 2 reflects DSO-related symptoms. Empty cells indicate loadings < 0.3 or non-primary loadings. | | | | | | | |

**
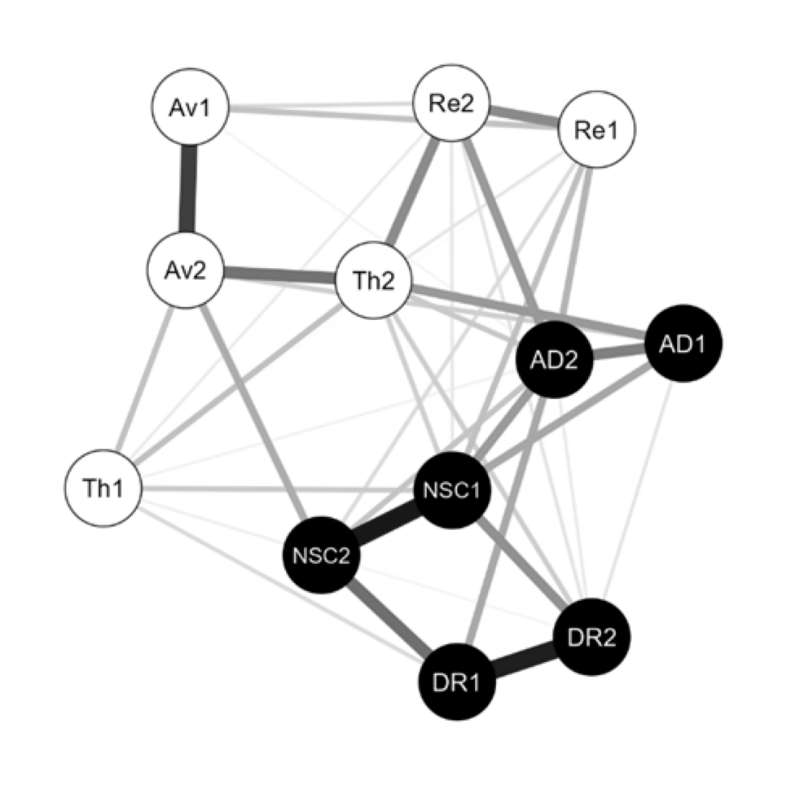
Network Analysis**

**Figure.**

*Network analysis of PTSD and DSO items*

**Alt Text:** Network diagram showing connections between PTSD and DSO symptoms based on partial correlations. Two main clusters emerge, one for PTSD (re-experiencing, avoidance, hypervigilance) and one for DSO (affective dysregulation, negative self-concept, disturbed relationships). Lines represent strength of association.

To further explore the structural organization of PTSD and DSO symptoms, we provide a network analysis by using the ITQ-CA symptom items. This approach afforded a nuanced understanding of the direct relationships between symptoms and highlights potential targets for future improvements. We estimate a regularized partial correlation network with the EBICglasso (extended Bayesian information criterion graphical least absolute shrinkage and selection operator) method. This method identifies the most robust associations between items by shrinking small edges to zero, reducing false positives, and improving interpretability.

Figure presents the resulting symptom network. Nodes represent individual symptom items from the ITQ-CA, and edges reflect the strength and direction of unique associations between them. The analysis reveals two highly connected symptom clusters, corresponding closely to the PTSD (re-experiencing, avoidance, hypervigilance) and DSO (affective dysregulation, negative self-concept, disturbed relationships) domains. Notably, the PTSD and DSO clusters are moderately interconnected, suggesting that while conceptually distinct, symptoms within these domains interact dynamically.

Within the DSO cluster, affective dysregulation items show the highest centrality (particularly item AD2: “When I am upset, it takes me a long time to calm down”), suggesting this symptom may play a pivotal role in maintaining broader CPTSD symptoms in children. Similarly, hypervigilance (Th2) emerged as a central node within the PTSD domain.
